# Quebec Eastern Township Regional Dementia Care Update after COVID. Reorganisation for the Quebec Ministerial Alzheimer program

**DOI:** 10.1002/alz.093027

**Published:** 2025-01-09

**Authors:** Guy Lacombe, Marie‐Pier Legresley, Michael Stiffel, Suzanne Gosselin

**Affiliations:** ^1^ Sherbrooke University, Sherbbrooke, QC Canada; ^2^ CIUSSS de l'Estrie‐CHUS, Sherbrooke, QC Canada; ^3^ CIUSSS de L'estrie‐CHUS, Sherbrooke, QC Canada; ^4^ CIUSSS de L'Estrie‐CHUS, Sherbrooke, QC Canada

## Abstract

**Background:**

In the province of Quebec, Canada, primary care management of Alzheimer’s disease is provided by the Family Medicine Group with the support of the Ministerial Initiative on Alzheimer’s Disease and the collaboration of specialized memory clinics. The COVID years have severely strained health resources in all areas, including cognitive intervention resources. We describe the reorganization of regional support in the Eastern Townships.

**Method:**

The results of a survey on the situation of care are shared with each of the family medicine groups involved in the Eastern Townships Alzheimer Plan, comparing the processes and results of each team to the provincial average. The difficulties and improvements proposed by each of the groups are noted and discussed with a view to continuous improvement.

**Result:**

A regional tour was necessary to re‐establish contact between the regional support resources (nurse and doctors of the memory clinic) and the Family Medicine Group. Nursing, medical, and pharmaceutical staff and social workers were met at each FMG. Interdisciplinary care models, local resources, leaderchip and development opportunities were discussed. Training needs have been identified. Demands to involve social workers and pharmacists are growing. The evolution of the clientele is striking, with a significant increase in the number of evaluations for patients with mild neurocognitive disorders.

**Conclusion:**

The presence of at least one experienced person at each FMG made it possible to reorganize the clinics after the staff changes related to the COVID period. The tour allows us to recreate the links that are essential to the development of expertise and to provide specialized support to the family medicine groups involved in the Alzheimer’s plan.

